# FIT and molecular tests versus colonoscopy: an economic evaluation of colorectal cancer screening in the United States

**DOI:** 10.3389/fonc.2026.1888178

**Published:** 2026-08-12

**Authors:** Yinyin Chang, Shujia Hao, Mao Mao

**Affiliations:** 1 Clinical Laboratories, Shenyou Bio, Zhengzhou, China; 2 Research & Development, SeekIn Inc, Shenzhen, China; 3 Research & Development, SeekIn Inc, San Diego, CA, United States; 4 Yonsei Song-Dang Institute for Cancer Research, Yonsei University, Seoul, Republic of Korea

**Keywords:** colonoscopy, colorectal cancer screening, economic evaluation, fecal immunochemical test (FIT), molecular tests

## Abstract

**Background:**

In 2021, the United States spent US$43.2 billion on five major cancer screenings, including US$27.5 billion for colorectal cancer (CRC). Colonoscopy screening (US$23.7 billion) accounting for 54.8% of total screening expenditures despite representing only 9.3% of initial tests. The high cost and resource intensity of colonoscopy as a primary CRC screening modality may constrain population-level implementation.

**Methods:**

We conducted a deterministic decision-analytic modeling study comparing five non-colonoscopy CRC screening strategies—fecal immunochemical test (FIT), two stool-based (Cologuard, ColoSense) and two blood-based (Shield, SimpleScreen) molecular tests—with initial colonoscopy screening in a simulated 2021 cohort of 9.2 million individuals. Positive non-colonoscopy results were assumed to trigger a follow-up colonoscopy. Primary analysis used an annual episode, a 10-year analysis estimated cumulative costs and colonoscopy demand based on intervals and adherence.

**Results:**

Clinical trial data demonstrated broadly similar CRC detection yield across five non-colonoscopy screening tests, however, blood-based tests showed limited ability to detect advanced precancerous lesions, whereas stool-based tests demonstrated superior performance. In the annual screening economic evaluation, colonoscopy detected 56,810 CRC cases and required 9.2 million procedures, with an average cost of US$2,571 per individual screened and a cost per CRC case detected of US$416,389. FIT detected 44,132 CRC cases, with a lower but directionally comparable yield to molecular tests (47,362-56,451 cases), required 298,544 follow-up colonoscopies, and had the lowest cost per individual screened (US$141) and per CRC case detected (US$29,318; 7.0% of colonoscopy). Replacing initial colonoscopy screening with FIT reduce total screening costs from US$23.7 billion to US$1.3 billion (94.5% reduction), saving US$22.4 billion. In the 10-year analysis, FIT remained the lowest-cost strategy (US$6.6 billion versus US$23.7 billion for initial colonoscopy) with substantially lower cumulative colonoscopy demand (1.5 million versus 9.2 million for initial colonoscopy). Stool-based molecular tests cost more than FIT (US$18.7–21.5 billion), and blood-based tests even higher than initial colonoscopy costs (US$42.1–42.4 billion) despite lower colonoscopy demand.

**Conclusions:**

Across both annual and 10-year cumulative analyses, these findings describe the short-term budgetary and resource implications of FIT-first CRC screening strategy, including reduced colonoscopy demand and screening expenditures while maintaining a broadly comparable CRC detection yield.

## 1 Introduction

Currently, well-established screening methods are in use for several cancer types, including breast, cervical, colorectal, lung, oral, prostate, skin, and stomach. In the United States, the United States Preventive Services Task Force (USPSTF) recommends screening for four cancer types (breast, cervical, colorectal, and lung), which account for approximately 25% of all cancers. Screening for these four types of cancer allows the early detection of nearly 15% of all cancer cases (1, 2). A recent study revealed that the U.S. spent US$43.2 billion on screening to detect five cancer types (breast, cervical, colorectal, lung, and prostate) in 2021 (3). The largest expenditure driver was colonoscopy for colorectal cancer (CRC) screening, which cost US$23.7 billion, representing 54.8% of the overall screening cost despite comprising only 9.3% of initial screening tests. The high cost of cancer screening is largely driven by colonoscopy being used as a primary screening modality for CRC in the U.S.

Although colonoscopy is widely adopted as the primary screening modality for CRC due to its high diagnostic accuracy and ability to detect and remove precancerous lesions, its invasiveness, high procedural cost, and resource requirements may limit population-level uptake and sustainability (4). These limitations have prompted the development and increasing use of less invasive screening alternatives aimed at improving adherence and expanding screening coverage. Current guidelines from the USPSTF recommend several CRC screening modalities, including stool-based tests (e.g., fecal immunochemical test (FIT) and multi-target stool DNA testing (Cologuard)), endoscopic examinations, and radiologic imaging (5). In recent years, increasing attention has been directed toward molecular screening approaches, such as emerging stool RNA-based assays (e.g., ColoSense), as well as blood-based tests based on circulating tumor DNA (e.g., Shield) and multi-omics technologies (e.g., SimpleScreen) (6–9). Among them, Cologuard received U.S. FDA approval in August 2014 and has become one of the most widely used cancer screening tests in terms of annual revenue generation (3, 10). ColoSense, Shield, and SimpleScreen are more recently approved tests, with U.S. FDA authorization granted in May 2024, July 2024, and July 2026, respectively (11–13).

Despite their growing clinical use, these tests demonstrate substantial heterogeneity in diagnostic performance across studies and reflect important trade-offs between sensitivity, specificity, convenience, and cost. The existing literature primarily focuses on comparisons among a limited number of established CRC screening modalities, although some modeling studies have evaluated blood-based and stool-based tests alongside colonoscopy (14–17). Moreover, differences in assumptions regarding test performance and cost inputs across studies further limit cross-study comparability. Therefore, there remains a need for a comparative analysis that evaluates multiple CRC screening strategies under a consistent analytical framework at the population level, particularly with respect to their economic efficiency under standardized assumptions.

In this study, we conducted a comparative economic evaluation focusing on the diagnostic performance and economic impact of five CRC screening strategies, including stool-based (FIT, Cologuard, and ColoSense) and blood-based (Shield and SimpleScreen) tests. A simulated 2021 U.S. colonoscopy screening cohort was used as the analytic cohort to evaluate differences in detection yield, colonoscopy demand, total screening costs, and cost per CRC case detected, and to assess their potential population-level economic implications.

## 2 Materials and methods

### 2.1 Study design and population

This study is a modeling-based economic evaluation of CRC screening in the United States. The primary objective was to compare the economic and resource implications of alternative non-colonoscopy CRC screening strategies as potential alternatives to colonoscopy-based screening in a real-world U.S. screening population. Specifically, the analysis evaluated differences in CRC detection yield, screening-related costs, and colonoscopy requirements to assess the trade-offs between screening performance and healthcare resource utilization. The analysis used 2021 CRC screening utilization and cost data derived from a published U.S. national healthcare economic analysis (3).

A population of 9.2 million individuals who underwent screening colonoscopy in 2021 was simulated (3). According to the source report, this population included men and women eligible for cancer screening services with an A (adults aged 50–75 years) or B (adults aged 45–49 years) grade recommendation from the USPSTF. The source dataset represented screening utilization; therefore, the modeled population reflected individuals undergoing CRC screening rather than patients undergoing diagnostic evaluation for symptoms.

### 2.2 Screening strategies

Five CRC screening strategies were evaluated as potential alternatives to colonoscopy, including three stool-based tests (FIT, stool DNA test Cologuard, and stool RNA test ColoSense) and two blood-based tests (cfDNA-based test Shield and multi-omics-based test SimpleScreen).

FIT is designed to detect the presence of hidden blood (hemoglobin) in stool, which is a sign of CRC and advanced precancerous lesions. Fecal occult blood tests (FOBTs), including FIT, have a long history in CRC screening (18). In the U.S., dozens of FIT kits are registered under the Clinical Laboratory Improvement Amendments (CLIA) database (19). Cologuard analyzes a panel of DNA markers associated with colorectal neoplasia, such as BMP3, NDRG4, and KRAS, as well as for human hemoglobin, similar to FIT (6). ColoSense is a multi-target stool RNA test that integrates RNA-based biomarkers associated with colorectal neoplasia and a fecal hemoglobin (FIT-like) component (7). Shield is based on a 500kb panel that interrogates genomic alterations, aberrant methylation status, and fragmentomic patterns of cell-free DNA (cfDNA) using next-generation sequencing (NGS) (8). SimpleScreen is a multi-omics test based on cell-free DNA-derived epigenetic and fragmentation signals for colorectal cancer detection (9).

### 2.3 Model structure and analytical framework

A deterministic decision-analytic framework was used to compare screening outcomes, costs, and healthcare resource utilization across these strategies. The model incorporated predefined screening strategies, test performance parameters, disease prevalence, adherence assumptions, and costs were used to estimate screening outcomes and resource utilization. Downstream diagnostic pathways after colonoscopy, subsequent surveillance procedures, and long-term clinical management after negative screening results were not explicitly modeled because the analysis focused on an annual CRC screening cost perspective using 2021 utilization and cost data.

The primary analysis evaluated a single-year annual screening scenario based on the 2021 U.S. colonoscopy screening cohort. The simulated cohort included 9.2 million individuals who underwent screening colonoscopy in the United States in 2021. Therefore, all individuals in this cohort were considered to have completed the screening episode, and no additional adjustment for initial screening adherence was applied. For each screening strategy, screening outcomes were estimated using the underlying CRC prevalence and published test performance characteristics. The numbers of true-positive, false-positive, true-negative, and false-negative results were calculated for each strategy. For non-colonoscopy strategies, all positive screening results were assumed to trigger follow-up diagnostic colonoscopy, with an 80% adherence rate based on published estimates (20). Individuals with negative screening results were considered to have completed the screening pathway without additional diagnostic procedures within the modeled screening episode. For the colonoscopy strategy, colonoscopy was considered the definitive screening and diagnostic procedure. Annual outcomes included CRC detection yield, false-positive screening results, follow-up colonoscopy requirements, total screening costs, cost per individual screened, and cost per CRC case detected.

In addition to the primary annual analysis, a supplementary analysis estimated cumulative screening costs and colonoscopy utilization over a 10-year horizon to account for differences in recommended screening intervals across modalities. Screening intervals were based on established guideline recommendations where available, including annual FIT, stool DNA testing every 3 years, and colonoscopy every 10 years (5). For blood-based tests, for which guideline-recommended screening intervals are not yet established, a 3-year interval was assumed for modeling purposes.

In the first screening round, the cohort was defined as the 9.2 million individuals who underwent colonoscopy screening in 2021, and no additional adherence adjustment was applied. From the second year onward, repeat screening adherence was incorporated to estimate subsequent screening volumes, with adherence values of 50% for FIT and 65% for other non-colonoscopy modalities based on previously published studies (21–23). For non-colonoscopy screening strategies, adherence to follow-up colonoscopy after a positive screening result was assumed to be 80% based on previously reported estimates (20). Individuals who underwent colonoscopy after a positive non-colonoscopy screening result in the previous screening round were excluded from subsequent screening rounds until their next recommended screening interval, as colonoscopy was recommended to be repeated every 10 years. The outcomes of the 10-year analysis included cumulative total screening costs and cumulative colonoscopy requirements over the 10-year screening horizon. This analysis was designed to evaluate cumulative resource utilization and budget impact, and did not model CRC incidence or detection dynamics across repeated screening cycles.

### 2.4 Data source

Model inputs were obtained from published clinical studies, national healthcare utilization reports, and available cost sources. Key model parameters included screening utilization, disease prevalence, test performance characteristics, screening adherence, and unit costs.

The screening population and colonoscopy utilization were derived from a recent report summarizing 2021 screening costs across five major cancers (3). The prevalence of CRC (0.65%) and advanced precancerous lesions (7.6%) in this cohort was derived from previously published U.S. screening data and applied uniformly across all screening strategies, without stratification by individual risk factors, to preserve comparability in this population-level analysis (6).

The performance characteristics of five screening tests for CRC screening and precancerous lesion detection, including sensitivity, specificity, positive predictive value (PPV), and negative predictive value (NPV), were derived from published literature and clinical trials. Specifically, Cologuard and FIT were derived from the pivotal DeeP-C study (6), ColoSense were derived from the CRC-PREVENT study (7), Shield and SimpleScreen was derived from the ECLIPSE (8) and the PREEMET study (9), respectively. Colonoscopy performance parameters were derived from a previously published decision-analytic modeling study conducted for the U.S. Preventive Services Task Force (24).

Unit costs for FIT (US$57), Cologuard (US$651), and colonoscopy (US$2,571) were calculated as total expenditures divided by the number of tests, based on a published 2021 national report of screening volumes and expenditures (3). ColoSense (US$508) and Shield (US$1,495) pricing were based on Medicare reimbursement rates (25, 26). Because publicly available pricing information for SimpleScreen was unavailable, its cost was assumed to be equivalent to that of Shield (US$1,495), as both are blood-based screening tests.

### 2.5 Costing methodology

The screening cost analysis included total screening costs, cost per CRC case detected, and cost per individual screened. Costs included only direct screening test costs and follow-up colonoscopy costs. Other medical costs, including CRC treatment and colonoscopy-related complications, as well as indirect costs such as patient time and transportation costs, were not considered.

For the primary annual analysis, costs were calculated based on a single screening round in the 2021 cohort. For the colonoscopy strategy, total costs were calculated as the unit cost of colonoscopy multiplied by the simulated number of individuals screened (9.2 million). For non-colonoscopy strategies, total costs included screening test costs and follow-up colonoscopy costs after positive screening results, calculated as the unit cost of each test multiplied by the number screened plus the unit cost of colonoscopy multiplied by the number of follow-up colonoscopies performed after applying the assumed follow-up colonoscopy adherence rate. Cost per CRC case detected was calculated by dividing the total cost by the number of CRC cases detected for each strategy. Cost per individual was calculated by dividing the total cost by the number individuals screened (9.2 million).

For the supplementary 10-year analysis, cumulative costs were calculated by summing the costs incurred across all simulated screening rounds over the 10-year horizon. Screening rounds were determined according to the recommended screening intervals and repeat adherence assumptions described above. Costs were not discounted because the unit costs for FIT, Cologuard, and colonoscopy were derived from actual 2021 expenditures, and the Medicare reimbursement rates used for ColoSense and Shield already represent administratively determined payment levels. The cumulative costs reflected repeated screening expenditures and follow-up colonoscopy costs associated with positive non-colonoscopy screening results across the 10-year period.

### 2.6 Statistical analysis

All modelling and comparison analyses were performed using R (v3.5.3, RRID: SCR_001905). A comparative economic evaluation framework was implemented in R to simulate screening outcomes and estimate performance metrics. Sensitivity, specificity, and predictive values were calculated by the epiR (v0.9-99, RRID: SCR_021673) package.

## 3 Results

### 3.1 Annual volume and expenditure of cancer screening in the United States

In 2021, 98.8 million individuals in the United States underwent initial screening tests for five major cancers (breast, cervical, colorectal, lung, and prostate), with total screening expenditures of US$43.2 billion (3).

**Figure 1:**
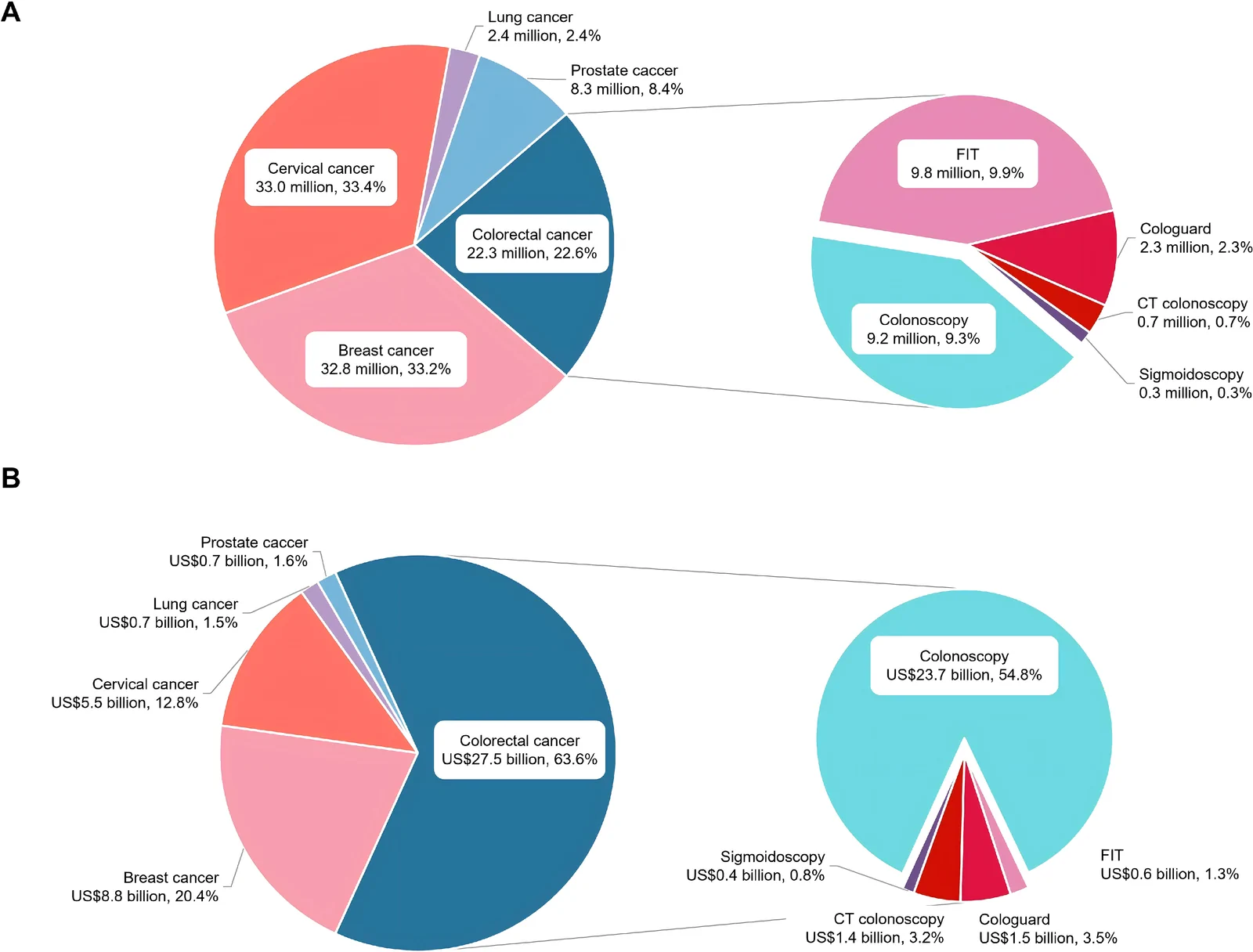
Volume and expenditure of cancer screening in the United States in 2021. **(A)** Volume. **(B)** Cost.

Among these, CRC screening accounted for 22.3 million tests (22.6%) overall (**Figure 1A**). FIT was the most frequently used modality (9.8 million, 9.9% of total screening volume), followed by colonoscopy (9.2 million, 9.3%). Other modalities included multitarget stool DNA testing (Cologuard; 2.3 million, 2.3%), CT colonography (0.7 million, 0.7%), and sigmoidoscopy (0.3 million, 0.3%). In contrast, **Figure 1B** demonstrates a very different distribution of screening expenditures. Colonoscopy alone accounted for US$23.7 billion, representing 54.8% of total national screening costs, despite constituting only 9.3% of total screening volume. Other screening modalities accounted for smaller shares of total expenditure, including FIT (US$0.6 billion, 1.3%) and Cologuard (US$1.5 billion, 3.5%), as well as CT colonography (US$1.4 billion, 3.2%) and sigmoidoscopy (US$0.4 billion, 0.8%). The high cost of cancer screening was largely driven using colonoscopy as the primary screening modality for CRC, with 9.2 million procedures performed in 2021 and an average cost of US$2,571 per colonoscopy (**Supplementary Table S1**).

### 3.2 Performance of five screening tests on CRC and precancerous lesion detections

**Figure 2:**
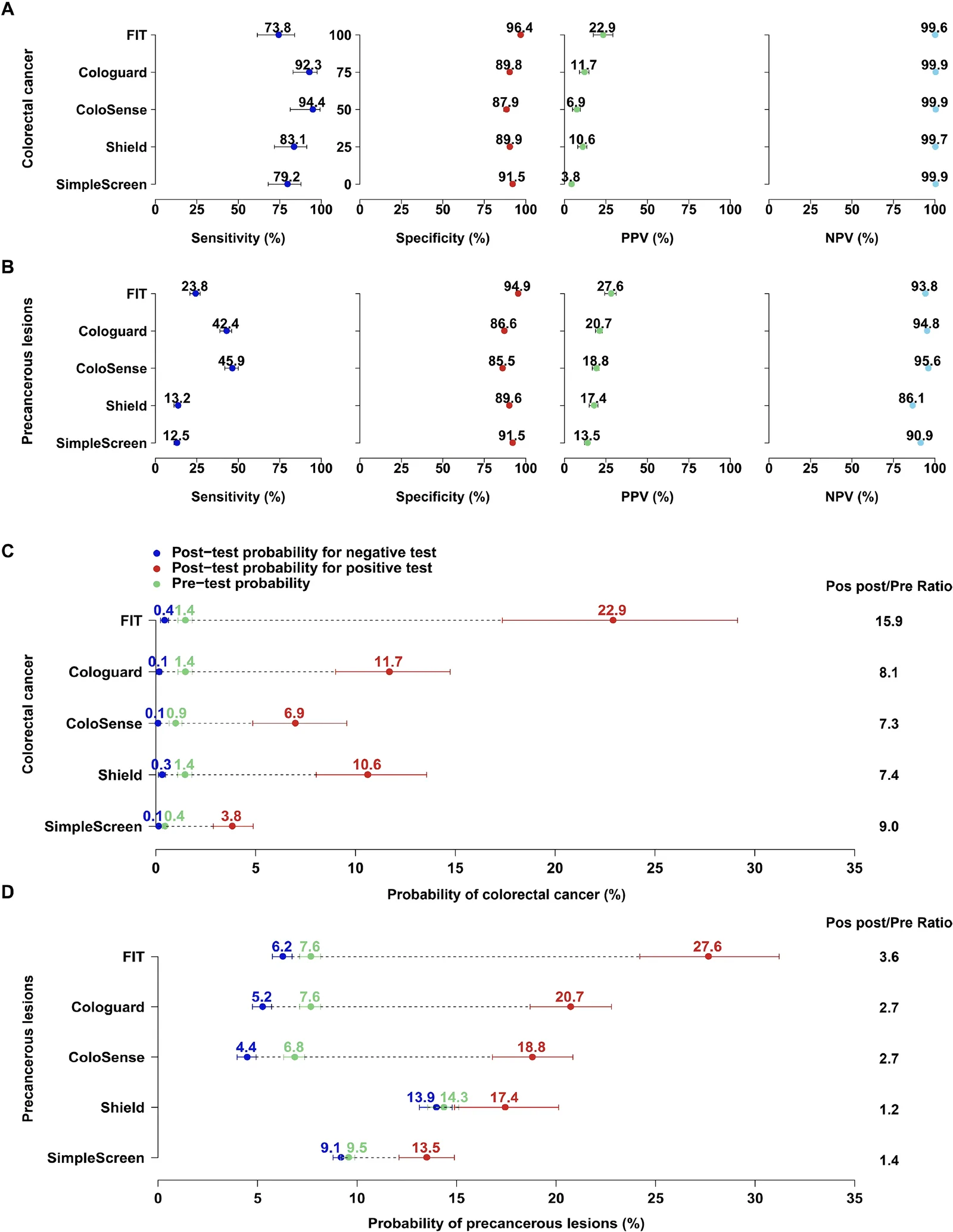
Performance and efficiency of five screening tests for colorectal cancer (CRC) and precancerous lesion detection. Sensitivity, specificity, positive predictive value (PPV), and negative predictive value (NPV) for CRC **(A)** and precancerous lesion **(B)**; Pre-test and post-test probabilities for CRC **(C)** and precancerous lesions **(D)** stratified by test results. Error bars indicate 95% CI. Pos post/Pre ratio: positive post-test probability/pre-test probability.

We compared the performance of five non-colonoscopy screening tests, sensitivities and specificities were estimated at different operating points and therefore described as comparative rather than strictly directly comparable across tests (**Figure 2**; **Supplementary Tables S2-S12**) (6–9). For CRC detection, FIT showed the highest specificity (96.4%) with lower sensitivity (73.8%), and demonstrated strong rule-in performance, with the highest PPV of 22.9%, whereas stool-based multi-target tests (Cologuard 92.3%, ColoSense 94.4%) achieved higher sensitivities but lower specificities (89.8% and 87.9%). Blood-based tests, Shield and SimpleScreen, showed intermediate sensitivities compared with stool-based tests (83.1% and 79.2%) with relatively high specificities (89.9% and 91.5%) (**Figures 2A, C**).

For advanced precancerous lesions, all evaluated screening tests showed limited sensitivity (**Figure 2B**). Colonoscopy demonstrated high sensitivity for detecting advanced precancerous lesions (85.0-95.0%) based on previously published data (24). In comparison, the non-colonoscopy screening tests showed lower sensitivity than colonoscopy, with blood-based tests showing the lowest performance. For positive post-test probabilities (equal to PPV), blood-based tests showed values close to baseline prevalence (pre-test prevalence), including Shield (17.4% vs 14.3%; post-test/pre-test ratio 1.2) and SimpleScreen (13.5% vs 9.5%; ratio 1.4). In contrast, stool-based tests showed higher positive post-test probabilities than baseline prevalence, with FIT 27.6% vs 7.6% (ratio 3.6), Cologuard 20.7% vs 7.6% (ratio 2.7), and ColoSense 18.8% vs 6.8% (ratio 2.7), indicating greater ability to identify individuals with advanced precancerous lesions (**Figure 2D**).

### 3.3 Annual analysis of screening outcomes, healthcare resource utilization, and cost of five screening tests and colonoscopy

In the simulated 9.2 million-person 2021 colonoscopy screening cohort, screening positivity and detection outcomes varied across strategies. FIT identified 44,132 true-positive cases, with broadly comparable detection yields across screening strategies, including SimpleScreen (47,362), Shield (49,694), Cologuard (55,195), ColoSense (56,451), and colonoscopy (56,810) (**Table 1**). Despite similar CRC detection yields across strategies, substantial differences were observed in false-positive results. FIT generated the fewest false-positive results (329,047), whereas molecular-based screening strategies generated higher numbers of false-positive results, ranging from 776,917 to 1,105,964 (**Table 1**).

**Table 1:** Simulated efficiency and costs of five screening tests vs colonoscopy in 2021 CRC screening.

Metric	Colonoscopy	FIT	Cologuard	ColoSense	Shield	SimpleScreen
Efficiency						
Number of true positives	56,810	44,132	55,195	56,451	49,694	47,362
Number of false positives	1,279,628	329,047	932,300	1,105,964	923,160	776,917
Number of true negatives	7,860,572	8,811,153	8,207,900	8,034,236	8,217,040	8,363,283
Number of false negatives	2,990	15,668	4,605	3,349	10,106	12,438
Positive predictive value (PPV)	4.3%	11.8%	5.6%	4.9%	5.1%	5.7%
Negative predictive value (NPV)	100.0%	99.8%	99.9%	100.0%	99.9%	99.9%
Positive likelihood ratio	6.8	20.5	9.0	7.8	8.2	9.3
Negative likelihood ratio	0.1	0.3	0.1	0.1	0.2	0.2
Pre-test probability	0.7%	0.7%	0.7%	0.7%	0.7%	0.7%
Post-test probability for positive test	4.3%	11.8%	5.6%	4.9%	5.1%	5.7%
Post-test probability for negative test	0.0%	0.2%	0.1%	0.0%	0.1%	0.1%
Number needed to screen (NNS)	162	208	167	163	185	194
Number needed to perform colonoscopy	9,200,000	298,544	789,997	929,932	778,283	659,423
Cost (US$)						
Total	23.7 billion	1.3 billion	8.0 billion	7.1 billion	15.8 billion	15.4 billion
Per CRC case identified	416,389	29,318	145,293	125,293	317,044	326,203
Per individual screened	2,571	141	872	769	1,713	1,679

The differences in screening outcomes translated into substantial variation in downstream colonoscopy demand. For non-colonoscopy strategies, follow-up colonoscopy requirements were estimated by applying an 80% adherence rate to diagnostic colonoscopy after positive screening results, including both true-positive and false-positive findings. FIT required 298,544 follow-up colonoscopies, which is markedly lower than that of stool- and blood-based molecular tests, including SimpleScreen (659,423), Shield (778,283), Cologuard (789,997), and ColoSense (929,932), and substantially lower than primary colonoscopy screening (9.2 million procedures) (**Table 1**).

From an economic perspective, FIT had the lowest cost per individual screened (US$141), representing 5.5% of the corresponding cost for colonoscopy (US$2,571) (**Table 1**). Compared with FIT, all molecular screening tests incurred substantially higher cost per individual screened, including ColoSense (US$769), Cologuard (US$872), SimpleScreen (US$1,679), and Shield (US$1,713) (**Table 1**). At the same time, FIT also achieved the lowest cost per CRC case detected (US$29,318), representing 7.0% of the cost of colonoscopy (US$416,389). Compared with other strategies, cost per CRC case detected was substantially higher for ColoSense (US$125,293), Cologuard (US$145,293), Shield (US$317,044), and SimpleScreen (US$326,203) (**Table 1**).

Overall, replacing primary colonoscopy screening with alternative strategies in the 9.2 million individuals currently undergoing colonoscopy screening was projected to substantially reduce annual CRC screening expenditures. FIT reduced total screening costs from US$23.7 billion to US$1.3 billion (94.5% reduction), resulting in US$22.4 billion in projected savings. Other strategies also reduced expenditures, including ColoSense to US$7.1 billion (70.0% reduction), Cologuard to US$8.0 billion (66.2% reduction), SimpleScreen to US$15.4 billion (35.0% reduction), and Shield to US$15.8 billion (33.3% reduction).

### 3.4 Ten-year cumulative cost and resource utilization analysis

**Figure 3:**
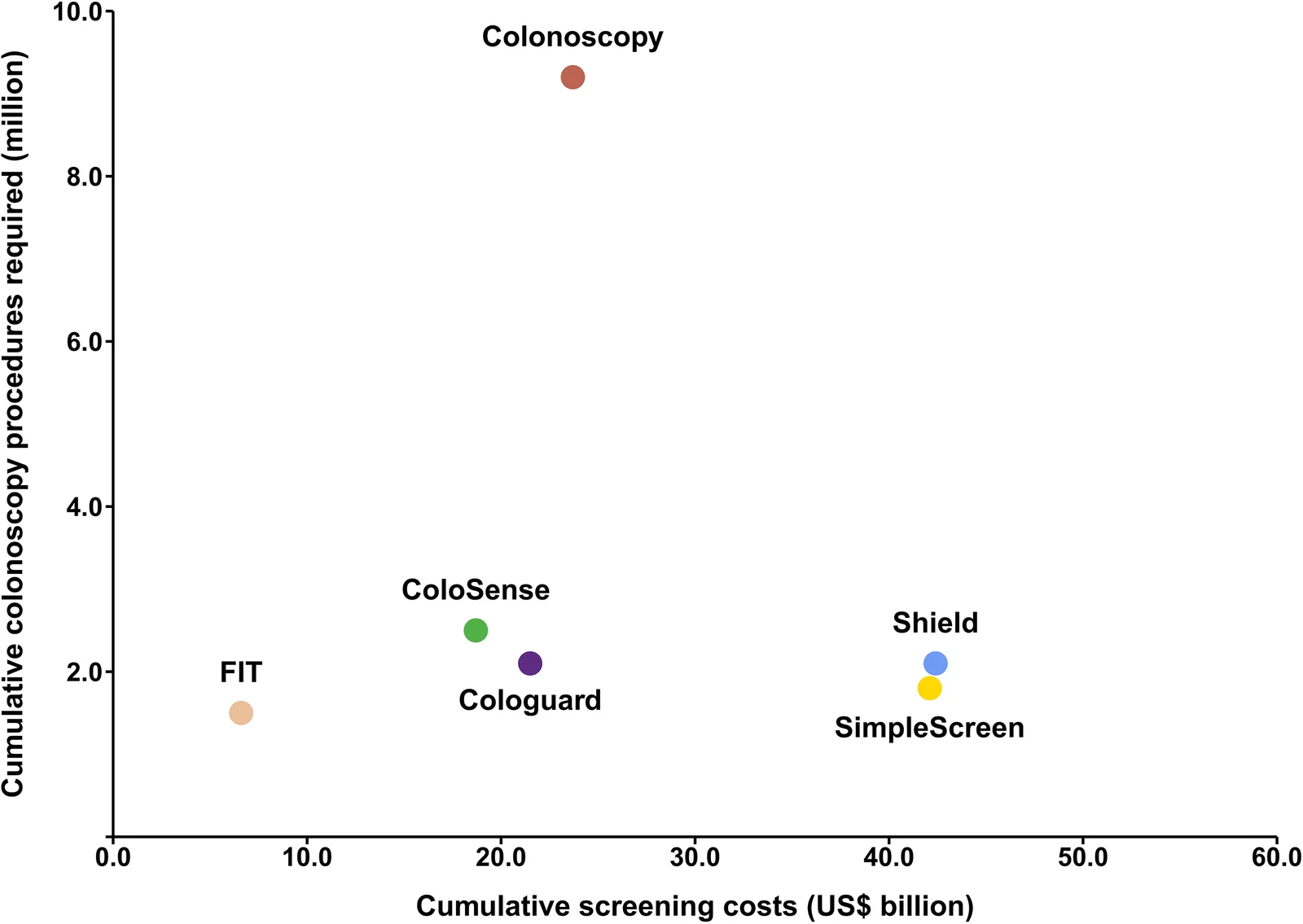
10-year cumulative costs and colonoscopy demand across colorectal cancer (CRC) screening strategies.

In a simulated 10-year CRC screening analysis, incorporating guideline-recommended screening intervals and adherence assumptions described above, substantial differences were observed in cumulative screening costs across strategies (**Figure 3**; **Supplementary Tables S13-S17**). FIT was associated with the lowest cumulative screening cost among all evaluated strategies (US$6.6 billion), despite being performed annually. In comparison, initial colonoscopy screening performed once every 10 years incurred a cumulative cost of US$23.7 billion. Stool-based molecular strategies, including ColoSense (US$18.7 billion) and Cologuard (US$21.5 billion), showed intermediate costs, whereas blood-based molecular strategies, including Shield (US$42.4 billion) and SimpleScreen (US$42.1 billion), had higher cumulative costs than initial colonoscopy screening.

Differences in cumulative colonoscopy demand were also observed across screening strategies (**Figure 3**; **Supplementary Tables S13-S17**). Colonoscopy screening resulted in the highest number of colonoscopy procedures (9.2 million). Annual FIT screening reduced cumulative colonoscopy demand to 1.5 million procedures required primarily for follow-up of positive screening results. Molecular screening strategies also required fewer colonoscopies than initial colonoscopy screening, although the magnitude varied across modalities, including SimpleScreen (1.8 million), Cologuard (2.1 million), Shield (2.1 million), and ColoSense (2.5 million). These findings highlight a critical trade-off between screening strategy and healthcare resource utilization, with FIT associated with both lower cumulative costs and fewer required colonoscopy procedures.

## 4 Discussion

The economic burden of CRC screening in the U.S. is substantial. In 2021, CRC screening accounted for US$27.5 billion in expenditures, largely driven by colonoscopy, which represented a disproportionate share of total cancer screening costs (US$23.7 billion; 54.8% of total cancer screening expenditures) despite a relatively small proportion (9.3%) of screening volume (3). In this modeling-based economic evaluation, we assessed the diagnostic performance and economic impact of five non-colonoscopy CRC screening strategies, FIT and four recently developed stool- or blood-based molecular screening tests (Cologuard, ColoSense, Shield, and SimpleScreen). We demonstrate that substantial differences in screening-related costs and healthcare resource utilization across screening modalities despite broadly comparable CRC detection yield.

Published clinical trial data showed that differences in overall CRC detection yield were relatively modest across strategies, whereas performance divergence was more pronounced in precancerous lesion detection. Blood-based molecular tests showed limited sensitivity compared with stool-based assays, likely reflecting limited shedding of tumor-derived signals into circulation in early or preinvasive disease, whereas stool-based tests may better capture exfoliated neoplastic material directly from the colorectal lumen (27, 28). These biological differences are particularly relevant in population-based screening, where early lesion detection is critical for long-term cancer prevention rather than CRC case detection alone.

Using a simulated U.S. initial colonoscopy screening cohort of 9.2 million individuals in 2021, we found that stool-based and blood-based molecular tests exhibit distinct trade-offs between detection yield and screening costs, with FIT demonstrated the most favorable economic profile under the assumptions of this analysis, achieving the lowest cost per CRC case detected, representing 7.0% of colonoscopy and substantially lower than other comparators, while achieving a lower but comparable CRC detection yield across strategies. Replacing initial colonoscopy screening with FIT could reduce CRC screening expenditures by 94.5%, corresponding to savings of US$22.4 billion. These findings were consistent in the 10-year analysis, in which FIT remained the lowest-cost strategy despite annual testing, with cumulative costs of US$6.6 billion over 10 years compared with US$23.7 billion for initial colonoscopy performed at the recommended 10-year interval. Stool-based molecular strategies, including ColoSense (US$18.7 billion) and Cologuard (US$21.5 billion), had higher cumulative costs than FIT but remained lower than initial colonoscopy screening. In contrast, blood-based molecular strategies, including Shield (US$42.4 billion) and SimpleScreen (US$42.1 billion), incurred substantially higher cumulative costs than both FIT and initial colonoscopy screening.

Additionally, the reduced number of downstream colonoscopy procedures required under FIT-based strategies highlights the broader impact of screening choice on healthcare resource utilization. Mounting evidence confirms that colonoscopy resources are already under significant strain in the U.S., where low-value endoscopic procedures contribute to prolonged waiting lists and case backlogs (29, 30). This reduction in demand is particularly important in resource-constrained systems. In such setting, using FIT as an initial screening test followed by colonoscopy for positive cases can improve screening accessibility while substantially reducing colonoscopy demand.

This FIT-first screening strategy has been widely implemented in international CRC screening programs and is supported by prior evidence demonstrating favorable participation and economic value (31–33). For example, the United Kingdom and the Netherlands have adopted FIT-based screening strategies due to resource and facility constraints that precluded primary colonoscopy (31, 32). Similarly, the Dutch national screening program confirmed the real-world effectiveness of the FIT-first strategy, demonstrating high participation and detection rates while optimizing the FIT cut-off to balance yield and resources (33).

These findings provide insights into the potential economic value of different CRC screening strategies in the U.S. healthcare setting. Under the assumptions of this analysis, greater use of FIT-based screening strategies may offer opportunities to reduce screening expenditures and colonoscopy demand. The potential cost savings may allow more efficient allocation of healthcare resources to improve screening adherence, address healthcare access disparities, or fund other high-value preventive services, including supporting careful evaluation and implementation of multi-cancer early detection (MCED) programs, thereby enhancing overall public health outcomes.

Several limitations of this study should be acknowledged. First, the analysis was based on a simulated cohort and published parameter inputs rather than individual-level real-world data, which may introduce uncertainty into the estimates of test performance and disease prevalence. Second, although the model incorporated a 10-year time horizon with strategy-specific screening intervals, it focused on cumulative screening costs and colonoscopy demand and did not model full lifetime screening trajectories or long-term clinical outcomes. Costs were not discounted because the analysis was designed as a budget impact evaluation. Third, the primary economic endpoints were screening-related costs and cost per CRC case detected, and the analysis did not include downstream healthcare costs such as CRC treatment, disease management, surveillance colonoscopies, colonoscopy-related complications, or the clinical and psychological consequences of false-positive results. This metric does not account for life-years or QALYs gained and should be interpreted as a short-term cost per case detected rather than a formal cost-effectiveness ratio. Fourth, publicly available pricing information for SimpleScreen was unavailable, and its unit cost was assumed to be equivalent to Shield. Changes in SimpleScreen pricing or reimbursement rate could alter its relative economic performance. Finally, key inputs such as CRC and advanced precancerous lesion prevalence were applied uniformly at the population level without stratification by individual risk factors, and the model did not incorporate the long-term preventive benefit of higher precancerous lesion sensitivity with colonoscopy. Consequently, this budget impact framework does not capture the long-term economic benefits of cancer prevention achieved by colonoscopy detection and removal of advanced precancerous lesions. Future studies that integrate individual-level data, longitudinal screening trajectories, cancer prevention outcomes, and comprehensive downstream costs will be needed to complement this economic evaluation and provide a more complete assessment of the long-term clinical and economic value of alternative CRC screening strategies.

This analysis extends prior work by using the most recent U.S. cancer screening volumes and expenditures, and by incorporating newly approved non-invasive CRC screening tests (ColoSense, Shield, and SimpleScreen) into a unified decision-analytic framework. Unlike many previous evaluations that considered only one or two non-colonoscopy options, this study compares multiple stool- and blood-based tests against colonoscopy under a consistent set of performance and cost assumptions at the population level.

In conclusion, under the assumptions of both cross-sectional and 10-year analyses, FIT represented the most economically efficient strategy among the evaluated screening strategies, with the lowest cost per CRC case detected and the greatest reduction in colonoscopy volume. These findings suggest that FIT-based screening strategies may represent a pragmatic approach to improve the economic value of CRC screening, relieve colonoscopy capacity constraints, and create budgetary space for other high-value preventive interventions.

## Data Availability

The original contributions presented in the study are included in the article/**Supplementary Material**. Further inquiries can be directed to the corresponding author.
