# Supplementary material for: FIT and molecular tests versus colonoscopy: an economic evaluation of colorectal cancer screening in the United States
Source: Front Oncol. 2026 Aug 12;16:1888178. doi: 10.3389/fonc.2026.1888178 (PMC13506431; doi:10.3389/fonc.2026.1888178)
Supplement: Supplementary file 1 [file Table1.docx]

*Supplementary Tables*

**FIT and molecular tests versus colonoscopy: an economic evaluation of colorectal cancer screening in the United States**

[Supplementary Table 1. Volume, total cost, and cost per cancer screening test in the United States in 2021.	1](#_Toc1636)

[Supplementary Table 2. Performance and efficiency of five screening tests on CRC and precancerous lesion detection.	2](#_Toc5591)

[Supplementary Table 3. CRC screening performance of FIT.	3](#_Toc7759)

[Supplementary Table 4. CRC screening performance of Cologuard.	3](#_Toc7008)

[Supplementary Table 5. CRC screening performance of ColoSense.	4](#_Toc28655)

[Supplementary Table 6. CRC screening performance of Shield.	4](#_Toc8556)

[Supplementary Table 7. CRC screening performance of SimpleScreen.	5](#_Toc2085)

[Supplementary Table 8. Precancerous lesions screening performance of FIT.	5](#_Toc2136)

[Supplementary Table 9. Precancerous lesions screening performance of Cologuard.	6](#_Toc14550)

[Supplementary Table 10. Precancerous lesions screening performance of ColoSense.	6](#_Toc27015)

[Supplementary Table 11. Precancerous lesions screening performance of Shield.	7](#_Toc20640)

[Supplementary Table 12. Precancerous lesions screening performance of SimpleScreen.	7](#_Toc12484)

[Supplementary Table 13. 10-year cumulative costs and colonoscopy demand across CRC screening strategies	8](#_Toc3391)

[Supplementary Table 14. Annual and cumulative screening population over 10 years analysis horizon.	8](#_Toc2439)

[Supplementary Table 15. Annual and cumulative follow-up colonoscopy utilization over the 10-year analysis horizon.	9](#_Toc1586)

[Supplementary Table 16. Screening positivity rates used for colonoscopy demand estimation	9](#_Toc1946)

[Supplementary Table 17. Annual and cumulative screening costs over the 10-year analysis horizon.	10](#_Toc31064)

# Supplementary Table 1. Volume, total cost, and cost per cancer screening test in the United States in 2021.

|  | **Total** | **Breast**  **cancer** | **Cervical**  **cancer** | **Lung**  **cancer** | **Prostate**  **cancer** | **Colorectal cancer** | | | | |
| --- | --- | --- | --- | --- | --- | --- | --- | --- | --- | --- |
|  |  |  |  |  |  | **Colonoscopy** | **FIT** | **Cologuard** | **CT colonography** | **Sigmoidoscopy** |
| **Number of individuals screened (millions)** | 98.8 | 32.8 | 33.0 | 2.4 | 8.3 | 9.2 | 9.8 | 2.3 | 0.7 | 0.3 |
| **Total cost (US$, millions)** | 43,169 | 8,802 | 5,542 | 656 | 702 | 23,655 | 560 | 1,497 | 1,402 | 353 |
| **Cost per individual screened (US$)** |  | 268 | 168 | 273 | 85 | 2,571 | 57 | 651 | 1,894 | 1,103 |

# Supplementary Table 2. Performance and efficiency of five screening tests on CRC and precancerous lesion detection.

|  | **CRC** | | | | | **Precancerous lesions** | | | | |
| --- | --- | --- | --- | --- | --- | --- | --- | --- | --- | --- |
|  | **FIT** | **Cologuard** | **ColoSense** | **Shield** | **SimpleScreen** | **FIT** | **Cologuard** | **ColoSense** | **Shield** | **SimpleScreen** |
| **Performance** |  |  |  |  |  |  |  |  |  |  |
| Sensitivity | 73.8% | 92.3% | 94.4% | 83.1% | 79.2% | 23.8% | 42.4% | 45.9% | 13.2% | 12.5% |
| Specificity | 96.4% | 89.8% | 87.9% | 89.9% | 91.5% | 94.9% | 86.6% | 85.5% | 89.6% | 91.5% |
| **Efficiency** |  |  |  |  |  |  |  |  |  |  |
| Positive predictive value (PPV) | 22.9% | 11.7% | 6.9% | 10.6% | 3.8% | 27.6% | 20.7% | 2.2% | 17.4% | 13.5% |
| Negative predictive value (NPV) | 99.6% | 99.9% | 99.9% | 99.7% | 99.9% | 93.8% | 94.8% | 100.0% | 86.1% | 90.9% |
| Positive likelihood ratio | 20.3 | 9.0 | 7.8 | 8.2 | 9.3 | 4.6 | 3.2 | 3.2 | 1.3 | 1.5 |
| Negative likelihood ratio | 0.3 | 0.1 | 0.1 | 0.2 | 0.2 | 0.8 | 0.7 | 0.6 | 1.0 | 1.0 |
| Pre-test probability | 1.4% | 1.4% | 0.9% | 1.4% | 0.4% | 7.6% | 7.6% | 6.8% | 14.3% | 9.5% |
| Post-test probability for positive test | 22.9% | 11.7% | 6.9% | 10.6% | 3.8% | 27.6% | 20.7% | 18.8% | 17.4% | 13.5% |
| Post-test probability for negative test | 0.4% | 0.1% | 0.1% | 0.3% | 0.1% | 6.2% | 5.2% | 4.4% | 13.9% | 9.1% |
| Number needed to screen (NNS) | 94 | 75 | 112 | 85 | 301 | 55 | 31 | 32 | 53 | 84 |
|  | | | | | | | | | | |

# Supplementary Table 3. CRC screening performance of FIT.

|  | **CRC +** | **CRC -** | **Total** |
| --- | --- | --- | --- |
| Test + | 48 | 162 | 210 |
| Test - | 17 | 4295 | 4312 |
| Total | 65 | 4457 | 4522 |
|  |  |  |  |
| Point estimates and 95% CIs: | | | |
| True prevalence | 0.0144 (0.0111, 0.0183) | | |
| Sensitivity | 0.7385 (0.6146, 0.8397) | | |
| Specificity | 0.9637 (0.9577, 0.9690) | | |
| Positive predictive value | 0.2286 (0.1736, 0.2914) | | |
| Negative predictive value | 0.9961 (0.9937, 0.9977) | | |
| Positive likelihood ratio | 20.3168 (16.4810, 25.0454) | | |
| Negative likelihood ratio | 0.2714 (0.1804, 0.4084) | | |

# Supplementary Table 4. CRC screening performance of Cologuard.

|  | **CRC +** | **CRC -** | **Total** |
| --- | --- | --- | --- |
| Test + | 60 | 455 | 515 |
| Test - | 5 | 4002 | 4007 |
| Total | 65 | 4457 | 4522 |
|  |  |  |  |
| Point estimates and 95% CIs: | | | |
| True prevalence | 0.0144 (0.0111, 0.0183) | | |
| Sensitivity | 0.9231 (0.8295, 0.9746) | | |
| Specificity | 0.8979 (0.8887, 0.9067) | | |
| Positive predictive value | 0.1165 (0.0901, 0.1474) | | |
| Negative predictive value | 0.9988 (0.9971, 0.9996) | | |
| Positive likelihood ratio | 9.0421 (8.0854, 10.1120) | | |
| Negative likelihood ratio | 0.0857 (0.0369, 0.1989) | | |

# Supplementary Table 5. CRC screening performance of ColoSense.

|  | **CRC +** | **CRC -** | **Total** |
| --- | --- | --- | --- |
| Test + | 34 | 456 | 490 |
| Test - | 2 | 3304 | 3306 |
| Total | 36 | 3760 | 3796 |
|  |  |  |  |
| Point estimates and 95% CIs: | | | |
| True prevalence | 0.0095 (0.0067, 0.0131) | | |
| Sensitivity | 0.9444 (0.8134, 0.9932) | | |
| Specificity | 0.8787 (0.8679, 0.8890) | | |
| Positive predictive value | 0.0694 (0.0485, 0.0956) | | |
| Negative predictive value | 0.9994 (0.9978, 0.9999) | | |
| Positive likelihood ratio | 7.7875 (6.9279, 8.7538) | | |
| Negative likelihood ratio | 0.0632 (0.0164, 0.2431) | | |

# Supplementary Table 6. CRC screening performance of Shield.

|  | **CRC +** | **CRC -** | **Total** |
| --- | --- | --- | --- |
| Test + | 54 | 457 | 511 |
| Test - | 11 | 4057 | 4068 |
| Total | 65 | 4514 | 4579 |
|  |  |  |  |
| Point estimates and 95% CIs: | | | |
| True prevalence | 0.0142 (0.0110, 0.0181) | | |
| Sensitivity | 0.8308 (0.7173, 0.9124) | | |
| Specificity | 0.8988 (0.8896, 0.9074) | | |
| Positive predictive value | 0.1057 (0.0804, 0.1356) | | |
| Negative predictive value | 0.9973 (0.9952, 0.9986) | | |
| Positive likelihood ratio | 8.2059 (7.1340, 9.4388) | | |
| Negative likelihood ratio | 0.1883 (0.1099, 0.3227) | | |

# Supplementary Table 7. CRC screening performance of SimpleScreen.

|  | **CRC +** | **CRC -** | **Total** |
| --- | --- | --- | --- |
| Test + | 57 | 1450 | 1507 |
| Test - | 15 | 15651 | 15666 |
| Total | 72 | 17101 | 17173 |
|  |  |  |  |
| Point estimates and 95% CIs: | | | |
| True prevalence | 0.0042 (0.0033, 0.0053) | | |
| Sensitivity | 0.7917 (0.6798, 0.8784) | | |
| Specificity | 0.9152 (0.9109, 0.9193) | | |
| Positive predictive value | 0.0378 (0.0288, 0.0487) | | |
| Negative predictive value | 0.9990 (0.9984, 0.9995) | | |
| Positive likelihood ratio | 9.3368 (8.2124, 10.6151) | | |
| Negative likelihood ratio | 0.2276 (0.1451, 0.3571) | | |

# Supplementary Table 8. Precancerous lesions screening performance of FIT.

|  | **Precancer +** | **Precancer -** | **Total** |
| --- | --- | --- | --- |
| Test + | 180 | 472 | 652 |
| Test - | 577 | 8695 | 9272 |
| Total | 757 | 9167 | 9924 |
|  |  |  |  |
| Point estimates and 95% CIs: | | | |
| True prevalence | 0.0763 (0.0711, 0.0817) | | |
| Sensitivity | 0.2378 (0.2079, 0.2698) | | |
| Specificity | 0.9485 (0.9438, 0.9529) | | |
| Positive predictive value | 0.2761 (0.2421, 0.3121) | | |
| Negative predictive value | 0.9378 (0.9327, 0.9426) | | |
| Positive likelihood ratio | 4.6181 (3.9555, 5.3917) | | |
| Negative likelihood ratio | 0.8036 (0.7720, 0.8365) | | |

# Supplementary Table 9. Precancerous lesions screening performance of Cologuard.

|  | **Precancer +** | **Precancer -** | **Total** |
| --- | --- | --- | --- |
| Test + | 321 | 1231 | 1552 |
| Test - | 436 | 7936 | 8372 |
| Total | 757 | 9167 | 9924 |
|  |  |  |  |
| Point estimates and 95% CIs: | | | |
| True prevalence | 0.0763 (0.0711, 0.0817) | | |
| Sensitivity | 0.4240 (0.3885, 0.4601) | | |
| Specificity | 0.8657 (0.8586, 0.8726) | | |
| Positive predictive value | 0.2068 (0.1869, 0.2278) | | |
| Negative predictive value | 0.9479 (0.9429, 0.9526) | | |
| Positive likelihood ratio | 3.1578 (2.8631, 3.4827) | | |
| Negative likelihood ratio | 0.6653 (0.6255, 0.7076) | | |

# Supplementary Table 10. Precancerous lesions screening performance of ColoSense.

|  | **Precancer +** | | **Precancer -** | **Total** |
| --- | --- | --- | --- | --- |
| Test + | | 278 | 1204 | 1482 |
| Test - | | 328 | 7074 | 7402 |
| Total | | 606 | 8278 | 8884 |
|  | |  |  |  |
| Point estimates and 95% CIs: | | | | |
| True prevalence | | 0.0682 (0.0631, 0.0737) | | |
| Sensitivity | | 0.4587 (0.4185, 0.4994) | | |
| Specificity | | 0.8546 (0.8468, 0.8621) | | |
| Positive predictive value | | 0.1876 (0.1680, 0.2084) | | |
| Negative predictive value | | 0.9557 (0.9507, 0.9603) | | |
| Positive likelihood ratio | | 3.1541 (2.8510, 3.4894) | | |
| Negative likelihood ratio | | 0.6334 (0.5883, 0.6819) | | |

# Supplementary Table 11. Precancerous lesions screening performance of Shield.

|  | **Precancer +** | **Precancer -** | **Total** |
| --- | --- | --- | --- |
| Test + | 147 | 698 | 845 |
| Test - | 969 | 5982 | 6951 |
| Total | 1116 | 6680 | 7796 |
|  |  |  |  |
| Point estimates and 95% CIs: | | | |
| True prevalence | 0.1432 (0.1354, 0.1511) | | |
| Sensitivity | 0.1317 (0.1124, 0.1530) | | |
| Specificity | 0.8955 (0.8879, 0.9027) | | |
| Positive predictive value (PPV) | 0.1740 (0.1490, 0.2012) | | |
| Negative predictive value (NPV) | 0.8606 (0.8522, 0.8687) | | |
| Positive likelihood ratio | 1.2606 (1.0676, 1.4885) | | |
| Negative likelihood ratio | 0.9696 (0.9463, 0.9934) | | |

# Supplementary Table 12. Precancerous lesions screening performance of SimpleScreen.

|  | **Precancer +** | **Precancer -** | **Total** |
| --- | --- | --- | --- |
| Test + | 321 | 2065 | 2386 |
| Test - | 2246 | 22306 | 24552 |
| Total | 2567 | 24371 | 26938 |
|  |  |  |  |
| Point estimates and 95% CIs: | | | |
| True prevalence | 0.0953 (0.0918, 0.0989) | | |
| Sensitivity | 0.1250 (0.1125, 0.1385) | | |
| Specificity | 0.9153 (0.9117, 0.9187) | | |
| Positive predictive value | 0.1345 (0.1211, 0.1489) | | |
| Negative predictive value | 0.9085 (0.9048, 0.9121) | | |
| Positive likelihood ratio | 1.4758 (1.3216, 1.6480) | | |
| Negative likelihood ratio | 0.9560 (0.9416, 0.9705) | | |

# Supplementary Table 13. 10-year cumulative costs and colonoscopy demand across CRC screening strategies

|  | **10-year cumulative screening cost (billion)** | **10-year cumulative colonoscopy demand (million)** |
| --- | --- | --- |
| **Colonoscopy** | 23.7 | 9.2 |
| **FIT** | 6.6 | 1.5 |
| **Cologuard** | 21.5 | 2.1 |
| **ColoSense** | 18.7 | 2.5 |
| **Shield** | 42.4 | 2.1 |
| **SimpleScreen** | 42.1 | 1.8 |

# Supplementary Table 14. Annual and cumulative screening population over 10 years analysis horizon.

| **Year** | **FIT** | **Cologuard** | **ColoSense** | **Shield** | **SimpleScreen** |
| --- | --- | --- | --- | --- | --- |
| **Year 1** | 9,200,000 | 9,200,000 | 9,200,000 | 9,200,000 | 9,200,000 |
| **Year 2** | 4,450,728 | 0 | 0 | 0 | 0 |
| **Year 3** | 4,378,514 | 0 | 0 | 0 | 0 |
| **Year 4** | 4,307,472 | 5,466,502 | 5,375,544 | 5,474,116 | 5,551,375 |
| **Year 5** | 4,237,582 | 0 | 0 | 0 | 0 |
| **Year 6** | 4,168,826 | 0 | 0 | 0 | 0 |
| **Year 7** | 4,101,186 | 5,161,389 | 5,022,362 | 5,173,109 | 5,292,738 |
| **Year 8** | 4,034,644 | 0 | 0 | 0 | 0 |
| **Year 9** | 3,969,181 | 0 | 0 | 0 | 0 |
| **Year 10** | 3,904,780 | 4,873,306 | 4,692,384 | 4,888,653 | 5,046,151 |
| **Total** | 46,752,912 | 24,701,198 | 24,290,290 | 24,735,877 | 25,090,264 |
| **Total (million)** | 46.8 | 24.7 | 24.3 | 24.7 | 25.1 |

Annual screening population = (initial screening population − cumulative number of individuals who completed follow-up colonoscopy in previous screening rounds) × repeat screening adherence.

# Supplementary Table 15. Annual and cumulative follo**w**-up colonoscopy utilization over the 10-year analysis horizon.

| **Year** | **FIT** | **Cologuard** | **ColoSense** | **Shield** | **SimpleScreen** |
| --- | --- | --- | --- | --- | --- |
| **Year 1** | 298,544 | 789,997 | 929,932 | 778,283 | 659,423 |
| **Year 2** | 144,428 | 0 | 0 | 0 | 0 |
| **Year 3** | 142,085 | 0 | 0 | 0 | 0 |
| **Year 4** | 139,779 | 469,404 | 543,358 | 463,088 | 397,903 |
| **Year 5** | 137,511 | 0 | 0 | 0 | 0 |
| **Year 6** | 135,280 | 0 | 0 | 0 | 0 |
| **Year 7** | 133,085 | 443,204 | 507,658 | 437,624 | 379,365 |
| **Year 8** | 130,926 | 0 | 0 | 0 | 0 |
| **Year 9** | 128,802 | 0 | 0 | 0 | 0 |
| **Year 10** | 126,712 | 418,467 | 474,304 | 413,560 | 361,690 |
| **Total** | 1,517,152 | 2,121,073 | 2,455,252 | 2,092,556 | 1,798,381 |
| **Total (million)** | 1.5 | 2.1 | 2.5 | 2.1 | 1.8 |

Annual follow-up colonoscopy demand = annual screening population × screening positivity rate × follow-up colonoscopy adherence.

10-year cumulative colonoscopy demand = sum of annual colonoscopy demand over the 10-year analysis horizon.

# Supplementary Table 16. Screening positivity rates used for colonoscopy demand estimation

| **Strategy** | **Positive screening results**  **(true-positive + false-positive)** | **Screened population** | **Positivity rate** |
| --- | --- | --- | --- |
| FIT | 373,180 | 9,200,000 | 4.1% |
| Cologuard | 987,496 | 9,200,000 | 10.7% |
| ColoSense | 1,162,415 | 9,200,000 | 12.6% |
| Shield | 972,854 | 9,200,000 | 10.6% |
| SimpleScreen | 824,279 | 9,200,000 | 9.0% |

Positivity rate was calculated as the number of positive screening results (true-positive plus false-positive results) divided by the total screened population.

# Supplementary Table 17. Annual and cumulative screening costs over the 10-year analysis horizon.

| **Year** | **FIT** | **Cologuard** | **ColoSense** | **Shield** | **SimpleScreen** |
| --- | --- | --- | --- | --- | --- |
| **Year 1** | 1,293,856,333 | 8,019,519,772 | 7,072,921,158 | 15,755,121,764 | 15,449,508,932 |
| **Year 2** | 625,935,066 | 0 | 0 | 0 | 0 |
| **Year 3** | 615,779,134 | 0 | 0 | 0 | 0 |
| **Year 4** | 605,787,983 | 4,765,078,426 | 4,132,695,686 | 9,374,495,964 | 9,322,393,088 |
| **Year 5** | 595,958,942 | 0 | 0 | 0 | 0 |
| **Year 6** | 586,289,378 | 0 | 0 | 0 | 0 |
| **Year 7** | 576,776,706 | 4,499,115,615 | 3,861,170,482 | 8,859,016,805 | 8,888,065,753 |
| **Year 8** | 567,418,378 | 0 | 0 | 0 | 0 |
| **Year 9** | 558,211,892 | 0 | 0 | 0 | 0 |
| **Year 10** | 549,154,782 | 4,247,997,516 | 3,607,484,950 | 8,371,882,504 | 8,473,973,591 |
| **Total** | 6,575,168,593 | 21,531,711,328 | 18,674,272,276 | 42,360,517,037 | 42,133,941,363 |
| **Total (billion)** | 6.6 | 21.5 | 18.7 | 42.4 | 42.1 |

Annual screening cost = annual screening population × screening test cost + annual follow-up colonoscopy demand × colonoscopy cost.

10-year cumulative cost = sum of annual screening costs over the 10-year analysis horizon
